# Amplitude modulated photostimulation for probing neuronal network dynamics

**DOI:** 10.1186/1471-2202-13-S1-P71

**Published:** 2012-07-16

**Authors:** Jonathan P Newman, Tatjana Tchumatchenko, Ming-fai Fong, Steve M Potter

**Affiliations:** 1 Department of Biomedical Engineering, Georgia Tech and Emory University, Atlanta, GA, 30332/ 30322, USA; 2 Center for Theoretical Neuroscience, Department of Neuroscience, Columbia University College of Physicians and Surgeons, New York, NY 10032-2695, USA; 3 Department of Physiology, Emory University School of Medicine, Atlanta, GA, 30303, USA

## 

Sensory input arrives in the cortex in the form of dynamic synaptic currents to populations of neurons. How cortical neurons encode and transmit these inputs ultimately determines the cognitive and behavioral response of the animal. Therefore, a number of theoretical studies have attempted to explain the cortical population response in model neuronal networks [1]. Yet, there are few experimental platforms for studying the dynamical rate responses in large living networks that match the manipulability of computational models. As a result, most experimental studies examining cortical input response properties are confined to independent or single neurons, e.g. [2].

**Figure 1:**
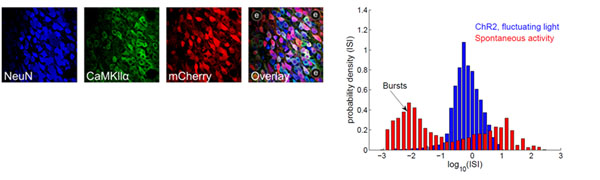
Figure 1. **(above)** DCN expression patterns of neuron-specific neuronal nuclear protien (NeuN), pyramidal specific CaMKIIa, and the ChR2 tag mCherry. The dots labeled ‘e’ are electrodes embedded in the MEA. **(right)** Inter-spike interval distribution recorded of single units in a DCN where pyramidal cells were expressing ChR2 subjected to continuous fluctuating blue light stimulation (blue) and during spontaneous, non-evoked, activity (red).

Optogenetic techniques in dissociated cultured networks (DCNs) that are grown on multielectrode arrays (MEAs) offer an opportunity to test key aspects of network response dynamics. However, controlling the spontaneous activity of DCNs and imposing a specific irregular activity pattern is challenging [3]. Here, we report that continuous fluctuating light stimulation has several major effects on DNCs that express Channelrhodopsin-2 (ChR2): (1) it inhibits network-wide bursting and leads to a unimodal interspike interval distribution of single units (figure 1) that resembles those observed *in vivo *[4]; (2) the single unit response to light fluctuations is temporally reliable and stable for prolonged periods of time; (3) the correlation between light and population firing can be > 0.6 for light fluctuations that have a 50 msec time constant; and (4) using a generalized linear framework we show that the linear response kernel can be used to predict the firing activity of neurons that are driven by light stimuli.

These results indicate that amplitude modulated optogenetic stimulation with LEDs is an efficient tool for providing correlated input currents to thousands of pyramidal cells embedded in DCNs while recording spiking activity in hundreds of individual neurons using an MEA. Because DCNs can be created with specified proportions of different cell types and allow easy pharmacological access, this technique opens a new level of control over living neuronal populations for testing theories of cortical response properties and small signal representation under different network parameter regimes.
